# Artificial Intelligence Applications in Implant Positioning, Dislocation Risk Prediction, and Surgical Indications in Orthopaedic Surgery

**DOI:** 10.3390/bioengineering13060610

**Published:** 2026-05-23

**Authors:** Mihai Emanuel Gherghe, Alex-Gabriel Grigore, Iosif-Aliodor Timofticiuc, Adelina-Elena Moise, Constantin-Adrian Andrei, Serban Dragosloveanu, Dana-Georgiana Nedelea, Łukasz Pulik, Catalin Anghel, Cristian Scheau, Romica Cergan

**Affiliations:** 1 Faculty of Medicine, The “Carol Davila” University of Medicine and Pharmacy, 050474 Bucharest, Romania; 2 “Foisor” Clinical Hospital of Orthopaedics, Traumatology and Osteoarticular TB, 021382 Bucharest, Romania; 3 Department of Orthopedics and Traumatology, Medical University of Warsaw, 02-005 Warsaw, Poland; 4 Department of Computer Science and Information Technology, “Dunărea de Jos” University of Galati, 800146 Galati, Romania

**Keywords:** artificial intelligence, machine learning, deep learning, orthopaedic surgery, arthroplasty, preoperative planning, outcome prediction

## Abstract

Background: Artificial intelligence (AI) is becoming increasingly integrated into orthopaedic surgery for tasks such as implant positioning, dislocation risk prediction, and surgical decision-making. However, the current evidence varies widely across anatomical regions and applications. Methods: A structured narrative review was conducted using PubMed and Web of Science Core Collection to identify studies applying machine learning or deep learning in orthopaedic procedures, focusing on parameters such as the anatomical region addressed, data types used, primary AI tasks, evaluation designs, and validation strategies. Reviews and meta-analyses were excluded. Study selection was summarized using a PRISMA-style flow diagram, and included studies were narratively synthesized according to anatomical region, AI task, imaging modality, validation strategy, and clinical relevance. Results: We identified three main application areas: (1) AI in imaging-driven planning and implant positioning, often linked with navigation or robotic systems; (2) postoperative evaluation related to implants; and (3) prediction of clinically relevant outcomes such as dislocation risk. The strongest evidence is found in hip arthroplasty, where AI improves measurement accuracy and workflow efficiency, whereas applications in knee, shoulder, and spine surgery are less developed and often supported by smaller studies. Although existing risk prediction models demonstrate good performance, their generalizability is hindered by limited external validation and inconsistent reporting. Conclusions: Overall, while AI shows significant promise in enhancing various aspects of orthopaedic surgery, stronger links between technical advancements and patient outcomes are needed. Future research should prioritize extensive validations, workflow-aware evaluations, failure analysis, and adherence to AI-specific reporting guidelines to facilitate safe and effective clinical implementation.

## 1. Introduction

Musculoskeletal disorders are among the leading causes of morbidity and disability worldwide [1]. Osteoarthritis and other degenerative joint diseases contribute substantially to disability and healthcare expenditures [2,3,4], and the prevalence of orthopaedic interventions, such as total joint arthroplasty, fracture fixation, and spinal instrumentation, has increased over the past few decades [5,6,7]. Arthroplasty volumes, in particular, have risen sharply. For example, the number of U.S. primary hip replacements increased by 156% and primary knee replacements by 136% between 1996 and 2019 [8]. Although these procedures remain highly successful, revision surgeries are costly and complex and are associated with high morbidity. The number of revision hip replacements increased by only 41% over the same period, whereas revision knee replacements rose by 147% [8,9].

With a rapid increase in procedural volumes and revision demands, the primary focus in orthopaedic surgery is to optimize technical factors that influence implant longevity and functional results. Achieving successful implant surgery involves several key considerations: accurate component placement [10,11,12], secure fixation [13,14], soft-tissue balancing [15], and suitable patient selection [16]. However, these factors are inherently variable and prone to human-related limitations, such as inter-surgeon differences [17], intraoperative judgment under time pressure [18], and difficulties in consistently translating preoperative plans into precise intraoperative execution [19,20]. These challenges have spurred growing interest in automated and technology-assisted solutions to enhance surgical planning, standardize intraoperative procedures, and minimize human variability.

Artificial intelligence (AI), including machine learning (ML) and deep learning (DL), is increasingly seen as a tool to support clinical decisions, improve risk assessments, and streamline complex workflows [21]. In orthopaedics, research on AI applications has surged recently, with ML and related techniques being explored to improve surgical planning, predict outcomes, and support decision-making, ultimately enhancing patient care [22]. For instance, convolutional neural networks (CNNs) can automatically detect anatomical landmarks on radiographs and determine alignment parameters to aid in total knee arthroplasty (TKA) planning [23]. Similarly, in hip arthroplasty, CNN-based models have demonstrated high accuracy in analyzing plain radiographs to identify implanted components, achieving validation accuracies over 95% and prospective accuracies above 90%, with quick inference suitable for clinical use [24]. AI algorithms can also be extended to three-dimensional (3D) systems [25,26], which show a higher rate of components placed within targeted alignment ranges than traditional 2D planning.

Postoperative complications such as dislocation, infection, and prolonged hospital stay significantly affect patient outcomes and healthcare costs [27,28]. DL models analyzing radiographs and clinical data have shown promise in predicting such complications. Rouzrokh et al. developed a DL model to forecast hip dislocation risk after primary total hip arthroplasty (THA) using postoperative radiographs, achieving a negative predictive value of 99.5%, thus effectively identifying low-risk patients [29]. Another study used MRI features and a random forest classifier to distinguish septic from aseptic failure in failed THA, with 92% sensitivity, 79% specificity, 89% positive predictive value, and 88% accuracy, with key predictive factors including bone edema, extracapsular edema, and synovitis [30]. Additionally, machine learning models have been employed to predict hospital length of stay (LOS) after THA/TKA by integrating demographic, laboratory, and surgical data [31].

With AI applications rapidly expanding in orthopaedics and the variety of published studies, a structured synthesis is necessary. This review covers three linked areas—implant positioning, dislocation risk prediction, and surgical indications—which collectively encompass the entire arthroplasty process. It assesses AI for preoperative planning (templating, landmark detection, alignment prediction), intraoperative guidance (navigation, robotics), postoperative monitoring (implant identification, loosening detection, infection classification), and risk prediction (dislocation, infection, length of stay, functional outcomes). The review evaluates model architectures, data sources, performance measures, validation methods, and clinical usefulness across hip, knee, and other orthopaedic subspecialties. It also discusses ethical issues, reporting standards, and possible future research directions. By gathering evidence from various studies, this review aims to provide clinicians and researchers with a critical overview of AI’s transformative role in orthopaedics and to identify responsible pathways for clinical adoption.

## 2. Materials and Methods

This article was designed as a structured narrative review rather than a systematic review or meta-analysis. A reproducible search strategy, predefined eligibility criteria, and PRISMA-style reporting were used to enhance transparency, while the synthesis remained narrative due to substantial heterogeneity across anatomical regions, imaging modalities, AI tasks, model designs, validation approaches, and reported outcomes.

### 2.1. Search Strategy

A literature search was performed in November 2025 in PubMed and the Web of Science Core Collection for original research articles on the use of artificial intelligence in orthopaedic surgery. A combination of Medical Subject Headings (MeSH), title/abstract terms, truncation, and Boolean operators was used to search PubMed. The Web of Science search was amended to use the Topic field, which searches titles, abstracts, author keywords and Keywords Plus. The search strategy was divided into three thematic parts: AI-related terms, anatomical/imaging/biomechanical analysis terms and orthopaedic surgery terms.

The first component targeted AI-related terms and technologies, including “Artificial Intelligence”, “Machine Learning”, “Deep Learning”, “Neural Networks, Computer”, and free-text variants such as “artificial intelligence”, “machine learning”, “deep learning”, “neural network*”, “convolutional neural network*”, “CNN”, “radiomics”, and “computer vision”. The second component focused on anatomical, morphological, biomechanical, and imaging-based analysis terms relevant to orthopaedic imaging and planning, including “anatomy”, “morphometry”, “morphology”, “biomechanics”, “Imaging, Three-Dimensional”, “3D imaging”, “CT-based analysis”, “radiographic analysis”, “implant position”, and “component position”. The third component limited results to orthopaedics using terms such as “orthopedic”, “orthopaedic”, “Orthopedic Procedures”, “Orthopedics”, “orthopedic surgery”, “orthopaedic surgery”, “bone surgery”, “joint surgery”, and “arthroplasty”.

The PubMed search string was: ((“Artificial Intelligence”[MeSH Terms] OR “Machine Learning”[MeSH Terms] OR “Neural Networks, Computer”[MeSH Terms] OR “artificial intelligence”[Title/Abstract] OR “machine learning”[Title/Abstract] OR “deep learning”[Title/Abstract] OR “neural network*”[Title/Abstract] OR “convolutional neural network*”[Title/Abstract] OR CNN[Title/Abstract] OR radiomics[Title/Abstract] OR “computer vision”[Title/Abstract]) AND (anatomy[Title/Abstract] OR morphometry[Title/Abstract] OR morphology[Title/Abstract] OR biomechanics[Title/Abstract] OR “Imaging, Three-Dimensional”[MeSH Terms] OR “3D imaging”[Title/Abstract] OR “CT-based analysis”[Title/Abstract] OR “radiographic analysis”[Title/Abstract] OR “implant position”[Title/Abstract] OR “component position”[Title/Abstract]) AND (“Orthopedics”[MeSH Terms] OR “Orthopedic Procedures”[MeSH Terms] OR orthopedic[Title/Abstract] OR orthopaedic[Title/Abstract] OR “orthopedic surgery”[Title/Abstract] OR “orthopaedic surgery”[Title/Abstract] OR “bone surgery”[Title/Abstract] OR “joint surgery”[Title/Abstract] OR arthroplasty[Title/Abstract])) NOT (review[Publication Type] OR meta-analysis[Publication Type] OR “systematic review”[Title/Abstract] OR “literature review”[Title/Abstract]), while the Web of Science Core Collection search string was: TS = ((“artificial intelligence” OR “machine learning” OR “deep learning” OR “neural network*” OR “convolutional neural network*” OR CNN OR radiomics OR “computer vision”) AND (anatomy OR morphometry OR morphology OR biomechanics OR “three-dimensional imaging” OR “3D imaging” OR “CT-based analysis” OR “radiographic analysis” OR “implant position” OR “component position”) AND (orthopedic OR orthopaedic OR “orthopedic surgery” OR “orthopaedic surgery” OR “orthopedic procedures” OR “orthopaedic procedures” OR “bone surgery” OR “joint surgery” OR arthroplasty)) NOT TS = (“systematic review” OR “meta-analysis” OR “literature review” OR review).

The combined search identified 397 records, 215 from PubMed and 182 from the Web of Science Core Collection. We removed 108 duplicate records and screened the titles and abstracts of 289 records. Of these, 195 were excluded and 94 reports were requested for retrieval. Six reports could not be retrieved. Eighty-eight full-text reports were assessed for eligibility. A total of 30 reports were excluded after full-text assessment for the following reasons: irrelevant outcome or indication, 9; no AI/ML/DL method applied, 6; non-orthopaedic or non-surgical focus, 5; insufficient methodology or validation detail, 8; and full text/language unavailable, 2. Finally, 58 studies were included in the review.

### 2.2. Eligibility Criteria

The eligible studies had to meet all the following criteria: original research articles, available as full text in English, concerned human orthopaedic surgical procedures or clinical relevant orthopaedic surgical planning, used an artificial intelligence, machine learning or deep learning method, and covered at least one of the following areas: surgical planning, anatomical or morphometric analysis, implant positioning, component evaluation, prediction of complications or outcomes, decision support, navigation, robotics, or postoperative evaluation of the implant.

The studies were excluded for the following reasons: review, systematic review, meta-analysis, editorial, letter, protocol, conference abstract without full text, animal-only studies, non-orthopaedic studies, non-surgical diagnostic studies without relevance to surgical planning or decision-making, studies without an AI/ML/DL component, and studies with insufficient methodological details to determine the type of model, task, or validation strategy.

### 2.3. Study Selection

**Figure 1:**
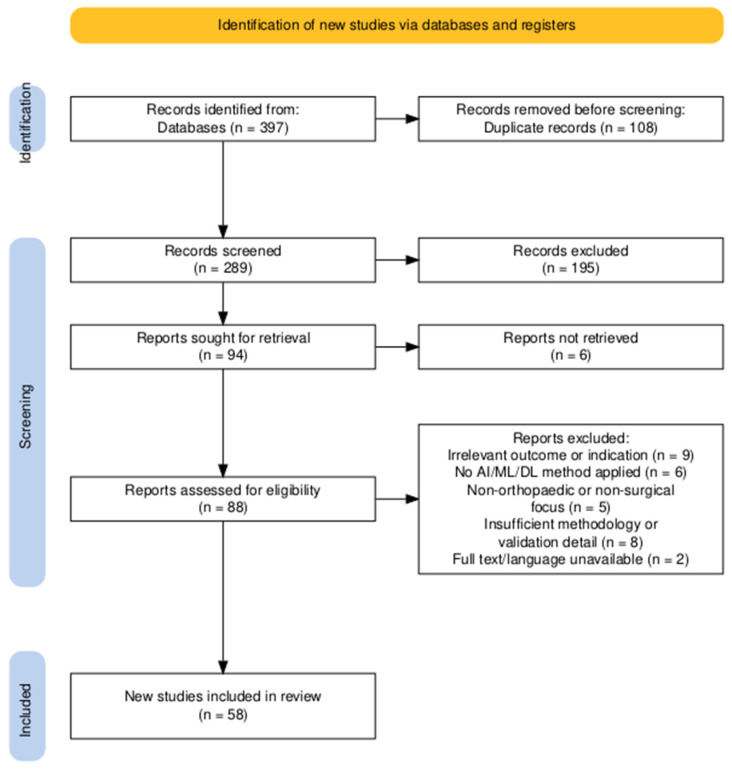
PRISMA-style flow diagram of study identification, screening, eligibility assessment, and inclusion.

Title and abstract screening was performed independently by two reviewers; after filtration, any disagreement about including certain articles was resolved by consensus between these two or, when consensus could not be reached, by a third senior reviewer. Potentially eligible articles were obtained for full-text review, which was again performed by two reviewers independently, with disagreements resolved through discussion among all authors. The reasons for full-text exclusion are recorded and summarized in the PRISMA-style flow diagram (Figure 1).

Studies unrelated to orthopaedic procedures or to artificial intelligence techniques for surgical planning, anatomical analysis, implant or component positioning, complication prediction, outcome prediction, or decision support were excluded during title and abstract screening. During the full-text evaluation, special attention was paid to whether the article reported an identifiable AI/ML/DL method, a clinically relevant orthopaedic surgical task, and sufficient methodological information to enable interpretation of the model’s purpose and validation approach.

Additional references were included when needed to support background explanations, define key concepts, and provide broader contextual or theoretical grounding. These sources were selected for their relevance and scientific credibility and were used to enhance the clarity, coherence, and interpretative depth of the review.

### 2.4. Data Extraction and Qualitative Appraisal

For each eligible study, we extracted key study characteristics: anatomical region, data type, AI task, model architecture, sample size, evaluation design, reference standard, reported performance metrics, validation strategy, and clinical relevance. Anatomical regions were grouped as pelvis/hip, knee, shoulder and spine. The data types included radiographs, CT scans, MRIs, fluoroscopy, ultrasound and/or clinical variables. AI tasks included segmentation, landmark detection, automatic measurement or templating, identification of implants, risk prediction, decision support, navigation-related planning, robotic workflow support, and postoperative evaluation of implants.

We also recorded the reference standard used in each study, such as expert annotation or measurement, intraoperative plan, postoperative imaging, or clinical outcome. When applicable, the reported performance metrics were extracted according to the task type, including the Dice similarity coefficient, accuracy, sensitivity, specificity, area under the receiver operating characteristic curve, mean absolute error, angular error, intraclass correlation coefficient, and other task-specific metrics. When reported, validation strategies were classified as internal split, cross-validation, temporal validation, external validation, or prospective/real-world assessment.

Due to the heterogeneity in study design, clinical application and AI task, formal meta-analyses and GRADE assessments were not conducted. Instead, the studies were qualitatively evaluated for methodological characteristics relevant to clinical translation such as sample size, single versus multicenter design, retrospective versus prospective design, patient-level data splitting during model development and validation, presence of external validation, type and reliability of the reference standard, reporting of calibration for prediction models, decision curve analysis where applicable, reporting of outliers or modes of failure, and evaluation of clinical workflow. For terminological clarity throughout this review, we use “AI-assisted” to describe applications in which AI input is implied in the surgeon’s decision-making process (automated measurements, suggestions for preoperative planning, risk outputs, which do not replace the human clinical judgement), and “AI-driven” to describe application in which AI is used autonomously (automatic implant identification, image segmentation, automated trajectory planning), which are later validated by surgical teams.

## 3. Results

The rapid development of artificial intelligence applications in orthopaedic surgical procedures has resulted in considerable heterogeneity in methodological approaches and clinical objectives. To establish an exhaustive examination of this expanding domain, studies were categorized by primary anatomical region: pelvic (hip and pelvis), knee, shoulder, and other orthopaedic subspecialties. This classification considers the biomechanical, anatomical, and clinical characteristics unique to each joint that influence the development and evaluation of AI models. Table 1 categorizes studies by anatomical area, identifies the predominant artificial intelligence techniques used, and outlines the primary clinical objectives for each category. This organizational framework enables systematic comparison of AI applications in preoperative planning, implant location, outcome prediction, and workflow automation across various orthopaedic specialties. The subsequent subsections examine each anatomical region comprehensively, including model architectures, data sources, performance measures, and translational relevance. They also identify the advantages, shortcomings, and future prospects of the employed methodologies for clinical integration.

### 3.1. Pelvic Region

AI methods have shown promise in augmenting preoperative planning and component placement in pelvic and hip surgery. For transsacral sacral fracture fixation, Kamer et al. utilized a CT-derived three-dimensional statistical shape model and machine learning to predict the personalized three-dimensional geometry of S1/S2 corridors [41]. Their AI-generated models illustrated the dimensions, morphology, and symmetry of the transsacral corridors S1 and S2, as well as the intended implant location, dimensions, orientations, and entry/exit points. Several studies have compared AI-assisted 3D planning with conventional approaches in total hip arthroplasty (THA). Huo et al. found that AI HIP software predicted acetabular cup and femoral stem diameters with accuracies of 74.6% and 71.2%, respectively, comparable to those of a 3D planning tool (71.2% and 76.3%) and significantly superior to those of a 2D template (40.7% and 49.2%) [40]. AI-assisted planning was far more efficient than manual planning with 3D software, taking around 3.9 min compared to 32.1 min. Lu et al. similarly reported that in cases of Crowe II–IV developmental hip dysplasia, AI-assisted 3D planning successfully predicted cup size in 59.2% of hips (compared with 30.2% with 2D planning) and stem size in 65.3% (compared with 41.9% with 2D planning), with AI-planned cases exhibiting a greater number of cups within the Lewinnek and Callanan safe zones, shorter operative times, less blood loss, and better correction of leg-length discrepancy [46]. Li et al. found that AI-3D planning achieved superior conformance rates compared to 2D planning, with 90.9% for the cup and 87.3% for the stem, versus 72.2% and 66.7%, respectively [44]. These findings consistently indicate that AI-driven 3D planning improves implant sizing and placement compared with traditional 2D templating. 

Nevertheless, AI does not universally outperform human control. Cardenas et al. [33] compared an AI-driven fluoroscopic navigation system with traditional human-controlled fluoroscopic navigation in primary total hip arthroplasty (THA). No significant difference was found: almost 95% of cups in both groups were located within the Lewinnek safe zone, and the precision of leg-length disparity was statistically similar. This indicates that AI can automate planning and measurement; however, its efficacy may not surpass that of an expert surgeon’s navigation when both use comparable imaging assistance.

AI has been utilized not just for planning but also for postoperative assessment. Ackermann et al. developed a fully automated method to assess outcomes after periacetabular osteotomy (PAO) [43]. They employed deep learning segmentation and image registration to determine the manipulation of fragments and the placement of screws. Their technique exhibited a screw-head localization error of approximately 1.3 mm and an axis deviation of approximately 1.1°, indicating high precision in establishing the optimal implant placement. Another study integrated a deep neural network with robotic imaging to assist in the placement of K-wires in the superior pubic ramus during pelvic fracture repair [42]. In cadaveric tests, the system identified the optimal wire trajectory within 2.8 ± 1.3 mm and 2.7 ± 1.8°, thereby reducing X-ray exposure by autonomously selecting the most advantageous perspective. These examples demonstrate that AI can assist with implant placement, both preoperatively and postoperatively, by automating measurements and identifying optimal trajectories.

An essential AI challenge for implant placement is identifying landmarks in radiographic images. Chan et al. developed a deep learning algorithm to identify osseous and implant landmarks on pelvic radiographs and fluoroscopic images [34]. Their program identified landmarks with comparable accuracy to trained human annotators and automatically estimated pelvic tilt, offset, leg length, and component inclination/anteversion. The capacity for real-time measurement may facilitate the verification of implant location during surgical procedures. A separate study demonstrated an automated 3D CT technique for quantifying femoral neck anteversion [49]. Using deep segmentation and landmark identification, their fully automated measures demonstrated accuracy comparable to typical manual approaches (intraclass correlations > 0.86, no significant differences, R > 0.9). Automated anteversion calculation can enhance preoperative planning by reducing observer variability.

Researchers have investigated the application of AI to forecast factors associated with postoperative instability after THA. Fontalis et al. investigated AI models incorporating spinopelvic characteristics to predict impingement, a marker of dislocation risk, in robotic total hip arthroplasty [37]. A gradient-boosting model employing tabular clinical data forecasted impingement with an accuracy of approximately 70.2%, flexion versus extension impingement with about 85% accuracy, and impingement type with nearly 73% accuracy. The integration of radiographs with a CNN did not improve accuracy, suggesting that the tabular planning data likely captured most of the information. Thus far, AI has only been able to make minor predictions regarding impingement in THA; however, there is potential for improvement. Similarly, spinopelvic motion influences stability. Zhao et al. developed a neural network to predict sitting pelvic tilt (PT) and sacral slope (SS) from standing lateral radiographs in healthy individuals [51]. Their most effective model achieved approximately 78–95% accuracy in predicting PT and SS. This indicates that machine learning can be employed to assess spinopelvic alterations, even when not within a total hip arthroplasty group. In THA patients, such forecasts may inform component positioning to avoid impingement or dislocation. Notably, no study has definitively demonstrated a correlation between AI predictions and actual postoperative dislocation rate.

Some AI research focuses on diagnostic or classification tasks that support surgical decision-making for hip pathologies. Uemura et al. developed a deep learning methodology to segment osteonecrotic lesions of the femoral head on MRI and classify them according to Steinberg grade automatically [48]. The network demonstrated exceptional segmentation accuracy (median Dice scores of ~0.95 for the femur and 0.89 for lesions) across 63 hips and accurately classified 93.7% of cases (weighted κ = 0.98). Automated grading may help surgeons decide between core decompression and arthroplasty for some patients. Hers et al. enhanced transformer-based segmentation (SegFormer3D) for hip ultrasonography in newborns with developmental dysplasia of the hip (DDH), thereby improving 3D cartilage segmentation efficacy [38] and demonstrating that early identification can address the need for surgical intervention.

Nonetheless, Table 2 concurrently presents the findings from all identified research related to the pelvic region.

### 3.2. Knee Joint

Artificial intelligence applications in knee surgeries primarily focus on preoperative planning and implant positioning evaluation. In total knee arthroplasty (TKA), accurate component sizing and alignment are crucial for functionality and long-term efficacy, with artificial intelligence being employed to improve these procedures. For example, Lan et al. investigated an AI-based three-dimensional preoperative planner alongside conventional two-dimensional templating [26]. The AI group more accurately assessed prosthetic dimensions and axial alignment angles, including valgus correction and hip–knee–ankle angles, than 2D planning, indicating statistically significant improvements. Patients scheduled with AI had superior short-term functional scores (WOMAC, AKS) at follow-up. Their study, while promising, had a limited sample size (60 knees) and lacked randomization, hence constraining its generalizability.

Additional studies have focused on deriving three-dimensional anatomy from conventional imaging. Factor et al. validated the efficacy of a commercial algorithm (RSIP XPlan.ai™—RSIP Vision, Jerusalem, Israel, https://www.rsipvision.com/2d-to-3d-joint-reconstruction-from-x-ray-images/, accessed on 8 May 2026) that generates 3D models of the femur and tibia from standard knee radiographs, thus eliminating the necessity for CT scans [56]. Their accuracy was reported to be under one millimeter, with global bone reconstruction errors of 0.9 mm and minimal local landmark errors around 0.5 mm. The axial alignment axes fluctuated by roughly 1–3°, similar to human variability. They assert that a commercial 2D-to-3D AI technique achieves clinically acceptable accuracy for TKA planning, while underscoring the need for additional validation on larger, more heterogeneous cohorts.

Several research studies have investigated implant sizing. Park et al. developed a multi-stage convolutional neural network on an extensive dataset of radiographs to autonomously measure the femoral and tibial components [62]. In approximately 40% of instances, the model precisely corresponded to the implanted size within a test set. In 89% of instances, it was within one size. An experienced surgeon could achieve 95–100% accuracy within one size, whereas AI operated at double the speed (~49 s compared to 98 s per case). Conversely, Yu et al. showed far higher accuracy, utilizing ResNet-101 with data augmentation on over 700 patients, achieving approximately 91% precise match for the femur and 87% for the tibia, with around 99% within one size [67]. Yu’s practically impeccable outcomes may be attributed to their controlled dataset (single surgeon/institution, significant augmentation), whereas Park’s multicenter methodology exhibited a broader “margin of error.” The varying outcomes demonstrate that the composition of a dataset and the architecture of AI significantly influence the efficacy of templating. Both works demonstrate that AI can automate labor-intensive templating; however, they also indicate that its accuracy currently falls short of that achieved by expert templating.

Another application of AI is the analysis of postoperative radiographs. Bonnin et al. developed X-TKA, a collection of 12 neural networks that autonomously assess the quality, implant size/orientation, alignment angles, and interface issues of standard knee radiographs [55]. The algorithms determined that the components were aligned with an average inaccuracy of approximately 1.7°, identical to the surgeon’s results. The surgeons’ proficiency in identifying issues at the interface between the implant and the bone was enhanced by around 5–12% with the utilization of X-TKA’s output, while the inter-surgeon consistency increased by a kappa value of +0.1 to +0.17. This suggests that AI could standardize the interpretation of post-TKA radiographs and assist doctors, although the study did not link this to patient outcomes.

Magg et al. employed deep learning to analyze 3D CT scans of tibial components in total knee arthroplasty under load to detect aseptic loosening [59]. They replaced a semi-automatic segmentation method with a fully automated CNN and found it maintained the same ability to distinguish between loose and fastened implants. In both cadaver and patient datasets, displacement parameters (translation/rotation) consistently demonstrated significant variations between loose and fixed cases within the automated procedure, in accordance with the prior method. The authors claim that full automation of implant displacement measurement is attainable without sacrificing diagnostic accuracy. This approach requires specialized CT scans under varus/valgus load, a process that is uncommon, hence restricting its immediate clinical application. 

No actual examples of AI models predicting knee implant dislocation were identified. True knee dislocation is rare after total knee arthroplasty, unlike hip arthroplasty, where the risk of dislocation is well-documented. Patellar instability constitutes a significant concern in the patellofemoral region. Certain AI research has examined the morphology of the trochlea, which is crucial for patellar tracking and may be associated with the risk of dislocation. A group of researchers, for example, trained a deep network to identify patellofemoral landmarks and assess joint geometry with an accuracy of approximately 2–3 mm [58]. Their model accurately reproduced critical angles and asymmetries in both healthy and arthroplasty knees, demonstrating that AI can assess anatomical features associated with instability. Deep learning has also been used to predict knee joint stresses and motions in the event of malrotated implants [68]. A deep network has been developed using simulated gait data from musculoskeletal models with different tibial-component misalignments, alongside four conventional machine learning approaches, to predict outcomes such as tibiofemoral contact forces and flexion angles [68]. The deep-learning algorithm surpassed all traditional techniques in precision. This early prediction facilitates the assessment of biomechanical outcomes across different implant rotations, potentially establishing “safe zones” for surgeons and robotic systems. This anatomical research may aid in the development of future risk models, mostly focused on predicting early risk of instability rather than dislocation. Overall, none of the included studies explicitly predict the risk of knee (or patellar) dislocation, indicating a deficiency in the literature. However, given the low incidence of this complication following total knee arthroplasty, its clinical relevance is limited. In routine practice, patellofemoral disorders more commonly manifest as anterior knee pain, maltracking, or lateral patellar overpressure, particularly in the absence of patellar resurfacing. Accordingly, AI-based evaluation of patellar geometry may be more effectively directed toward stratifying the risk of these outcomes, albeit this remains speculative. 

Artificial intelligence has not been widely employed in the selection of candidates for knee surgery. Sappey-Marinier et al. contend that AI could improve patient selection for TKA using predictive models; yet, current models are still “imprecise” [89]. In other words, robust AI algorithms for determining the appropriate timing for knee arthritis surgery are now lacking; physicians predominantly retain the decision-making responsibility. The literature we reviewed lacked any primary trials on AI-driven surgical indications for knee surgeries. The application of AI for knee surgery remains predominantly speculative. The aforementioned perspective suggests that future technologies may emerge; yet, at present, this field lacks validated applications and represents an unmet need.

Consequently, Table 3 presents all pertinent studies about AI models and tasks utilized in knee joint applications.

### 3.3. Shoulder Area

When it comes to shoulder surgery, AI tools and models primarily focus on segmentation for planning, implant identification and positioning, and outcome prediction. Kim et al. developed a CNN-based segmentation (nnU-Net) of rotator cuff structures using 2D MRI to facilitate the creation of a 3D model [72]. The 3D nnU-Net achieved remarkable accuracy with merely 56 cases (Dice ≈ 0.81 for tendon, 0.86 for muscle, and ≈0.98 for bone/cartilage). Yang et al. used DeepLabV3+ on shoulder CT to determine the volume of rotator cuff muscle and adipose tissue in preoperative and two-year follow-up scans [82]. The Dice scores were considerably improved (~0.928 pre-operative, ~0.916 at two years), and the mean surface error was under one millimeter. These experiments illustrate that deep CNNs can automate tedious manual segmentation with excellent accuracy, suggesting possibilities for virtual modeling in preoperative planning. However, both studies employed restricted datasets (comprising tens of cases) and specific criteria (rotator cuff arthropathy or repair), making generalizations (e.g., to varied pathologies or multi-institutional images) unproven.

Studies have shown that AI models can autonomously identify anatomical landmarks on shoulder radiographs. Shariatnia et al. trained a U-Net/EfficientNet model using roughly 1200 AP radiographs to detect the glenoid and inferior acromion landmarks and to compute the critical shoulder angle (CSA) and acromion index (AI) [77]. The mean absolute errors for CSA and AI were 1.68° and 0.03°, respectively, on the test set, including 93 images. This accuracy (~2°) parallels that of human evaluators and demonstrates that CNNs can reliably evaluate risk-predictive morphometrics on a large scale. This approach’s efficacy is rooted in its use of a significant, publicly accessible dataset (MURA) and its validation for extensive study applicability. A disadvantage is that it was exclusively taught on “normal” shoulders; its efficacy on postoperative or pathological conditions remains unknown. Additionally, CSA/AI metrics serve as risk indicators for rotator cuff tears or arthritis rather than direct indications for surgery, necessitating further research to integrate these automated measurements into clinical decision-making.

Artificial intelligence methods have been applied to the detection and positioning of implants. Sultan et al. proposed employing ensemble CNNs to differentiate various types of shoulder prostheses from radiographs [80]. A DenseNet/ResNet ensemble (DRE-Net) utilized in the analysis of 538 implant radiographs achieved an accuracy of 85.9% (F1 ≈ 0.85) in distinguishing between implant models. An improved IMFC-Net ensemble, assessed on an expanded dataset of 597 pictures, attained an accuracy of 89.1% [80]. Identifying implants can enhance revision planning using computerized “surgical indication” techniques for instrument selection. 

Yang et al. developed a convolutional neural network (CNN) to segment the reverse TSA glenosphere and identify its center on anteroposterior radiographs [81] to assess the implant’s position post-surgery. The model achieved an average Dice score of approximately 0.86 for segmentation, and its measurements (glenosphere medialization, inferiorization, etc.) exhibited an intraclass correlation of roughly 0.90–0.96 with human raters. In fact, the AI required approximately 2 s per image, thereby simplifying a challenging and error-prone task. The significant consensus indicates its potential efficacy in a clinical environment. Nonetheless, it was evaluated using optimal postoperative radiographs; hence, it may not perform as effectively with suboptimal images or abnormal anatomy. Spangenberg et al. introduced an extra planning phase by training a 3D deep learning algorithm on CT-derived shoulder reconstructions to predict the humeral head resection plane [78]. The predicted planes closely aligned with the surgeon’s assertions: the mean centroid error was approximately 1.4 mm, and the orientation error was around 3.9° in arthritic bones (much more favorable in non-arthritic bones). Errors of less than 5° are within the permissible range in surgical practice. The model’s strength lies in its “state-of-the-art” accuracy, even in the presence of osteophytes. Nonetheless, it was exclusively trained and evaluated on 62 shoulders, requiring further testing on additional shoulders. Incorporating such a tool into planning software might facilitate humeral osteotomy; however, regulatory issues must be resolved beforehand.

Machine learning algorithms have been trained for predicting postoperative outcomes, which pertains to surgical indications (for instance, determining when surgery is likely to be beneficial). Kumar et al. utilized XGBoost on 5774 shoulder arthroplasty patients (TSA and RTSA) to predict numerous outcomes (ASES, Constant score, range of motion, etc.) [73]. They compared a full model (291 pre-op features) vs. a pared-down 19-feature model. Both displayed roughly the same number of errors in their predictions (ASES MAE ~11.7 versus 12.0 points; other metrics were comparable). The model accurately identified the risk of clinical improvement with minimal input, demonstrating its user-friendliness during appointments. Nevertheless, MAEs ranging from 10 to 12 points indicate only moderate precision, limiting individual decision-making. The method requires substantial high-quality data and has only been evaluated internally within the organization. Rajabzadeh et al. utilized XGBoost on preoperative shoulder CT scans of 1057 patients to assess if deltoid muscle attributes improve outcome predictions [76]. They taught CNN to automatically divide the deltoid into parts and get measurements of volume, shape, and HU. Including these imaging features somewhat improved the 1–5 year outcome mean absolute errors in comparison to models lacking them. The volume and “flatness” of the deltoid were among the most significant predictors, surpassing other demographic characteristics. This demonstrates the utility of AI-generated anatomical features in decision-making processes. The advantages remained minimal, and the model was compatible solely with one implant method, lacking rotator cuff data at this stage. Overall, tabular AI (XGBoost) may categorize patients; yet, the existing models exhibit only moderate accuracy (AUC/accuracy ~0.8–0.9 as referenced) and require validation prior to their application in surgical decision-making.

Finally, AI-assisted fracture planning has emerged. Jeon et al. devised a procedure termed “AI-assisted reduction” for the management of 3- or 4-part proximal humerus fractures [71]. Their pipeline segmented the fracture fragments using deep learning algorithms and virtually reduced them through optimization. The AI model demonstrated significantly greater shape overlap with postoperative CT in comparison to manual reduction, achieving a mean Dice Similarity Coefficient (DSC) of 0.78 versus 0.69 (*p* < 0.001) and an Intersection over Union (IoU) of 0.65 versus 0.55. The quality scores for surgeons were marginally elevated (RQS ≈ 91.5 compared to 89.3, *p* = 0.045), while AI planning consumed around 1.4% of the time required by human approaches. This signifies substantial enhancements in efficiency. The study comprised only 20 cases and reproduced precisely controlled conditions. The human validity agreement (CVA) was approximately 82%, indicating that some surgeons perceived manual methods as comparable to AI. Prior to clinical application, additional testing, including various fracture types and imaging techniques, is required. 

These shoulder-focused AI studies demonstrate that modern algorithms (CNNs and ensemble methods) can match or surpass human performance in specific tasks. Such tools are promising in a clinical setting (speeding up workflows and measuring complex features), but they need to be tested in rigorous prospective trials. Table 4 shows all available data taken from identified papers about artificial intelligence models in shoulder surgery.

### 3.4. Spine

Recent methods employ machine learning (ML) and deep learning for activities associated with spine surgery, including surgical planning, implant guidance, and outcome prediction. Numerous studies have demonstrated that AI is able to effectively plan or assist in the placement of spinal hardware. Yang et al. employed a 3D U-Net convolutional network for the automated planning of thoracolumbar pedicle screws [86]. The Dice segmentation score exceeded 0.94, and 98.8% of the screw positions were classified as Gertzbein–Robbins A (no breach). This indicates that CNN-based algorithms can efficiently and precisely determine the trajectory of screws, nearly matching the proficiency of experts. Luchmann et al. developed an AI-driven fluoroscopic navigation system named X23D, which uses four X-ray images to construct a 3D model of the spine for the insertion of lumbar screws [84]. In an ex vivo comparison of 49 screws, X23D navigation exhibited a somewhat reduced breach rate (21% compared to 24% in the freehand control) and utilized less radiation on average (33.3 mGy against 49.5 mGy); however, the changes were not statistically significant. The findings of these assessments establish that AI-driven navigation can achieve accuracy similar to conventional techniques while using reduced radiation levels. Luchmann et al. assert that additional efforts are required to achieve “clinical-grade” accuracy [84]. The two strategies for implant positioning are distinct from one another. Yang et al. conducted training on over 1200 CT cases and employed established grading schemes to evaluate performance [86]. Luchmann et al. utilized a limited sample of cadavers to assess the efficacy of the approach within a simulated surgical workflow [84]. Both demonstrate that AI methodologies can increase the precision of spinal instrumentation; however, further research and prospective trials are necessary to validate their pragmatic utility.

Artificial intelligence has also been employed to forecast risks and outcomes in spinal surgery. Ye et al. examined 280 cervical spine cases (CT angiograms) to determine the probability of vertebral artery injury (VAI) during the insertion of the C2 pedicle screw [87]. They used various machine learning methods to analyze 15 patients and their anatomical characteristics. A neural network displayed superior performance, achieving an AUC of approximately 0.936 on validation. Explainable AI (SHAP) identified six significant characteristics that increase the likelihood of VAI, including the diameter of the pedicle and the elevated position of the vertebral artery. Wong et al. developed a support vector machine on preoperative MRI muscle measurements to forecast early adjacent-segment degeneration (ASD) following multilevel ACDF [85]. Their SVM model attained an accuracy of 96.7% (AUC 0.97) in identifying patients with early-onset ASD, with paraspinal muscle asymmetries, particularly fat asymmetry at C5, serving as the most significant markers. Increased accuracy scores demonstrate the potential of AI to identify patients at the greatest risk before surgery. Both studies utilized limited patient cohorts (280 for VAI and around 60 for ASD) and relied on historical data, making the assessment of the accuracy and transferability of the results important. Ye et al. and Wong et al. point out the necessity of testing their VAI model across several sites, with Wong et al. additionally noting that their limited sample size indicates a requirement for further research [85,87]. Although AI models appear capable of assessing the likelihood of problems, direct comparison is difficult due to the dissimilar data types (CT versus MRI) and methodologies (neural networks versus support vector machines).

Finally, AI assists in assessing the requirement for surgical intervention and evaluating the outcomes. Zheng et al. created a machine learning model using radiomics that can predict if a patient will have significant neurological recovery (JOA recovery rate ≥ 50%) after cervical laminoplasty. Three different methods were used to outline the spinal cord in three dimensions, showing that a support vector machine using data from the “narrowest segment” reached an area under the curve of about 0.885 [88]. The area under the curve of the integrated model increased to around 0.967 when radiomics were combined with four clinical factors, including smoking, diabetes, pre-operative JOA, and sagittal alignment. This was significantly greater than models that relied solely on clinical or imaging data. This conclusion indicates that AI could considerably influence surgical decisions by identifying patients who are likely to benefit from laminoplasty. Li et al. used an enhanced V-Net deep-learning segmentation algorithm of vertebral CT images to evaluate two interventions for osteoporotic compression fractures in the lumbar spine: kyphoplasty (PKP) and vertebroplasty (PVP) [83]. Their improved V-Net produced much better Dice coefficients than the standard U-Net or CNN models, permitting accurate creation of 3D vertebral models. These data demonstrated that PKP surpassed PVP in restoring the heights of the anterior, middle, and posterior vertebrae while reducing the kyphotic angle. It also offered enhanced analgesia and an increased incidence of “excellent or good” results. It is claimed that PKP is better for treating osteoporotic thoracolumbar fractures, highlighting the usefulness of AI-based imaging in assessing how well the surgery performs. Moreover, Li et al. recognize that their segmentation algorithm requires continued validation due to the small sample size and restricted fracture types [83].

Looking at different methodologies implemented in various studies shows both commonalities and distinctions. In jobs requiring predictions using tables, classical machine learning classifiers such as support vector machines and logistic regression are predominant. Deep convolutional neural networks (CNNs) and U-Nets are the predominant architectures for image-related tasks. Zheng et al. and Ye et al. looked at different methods and found that Support Vector Machines (SVMs) worked best for labeling radiomics, while neural networks were better at evaluating VAI risk [68,87]. Conversely, Yang et al. exclusively examined a 3D U-Net for surgical planning [86]. Various evaluation measures are also employed. For instance, binary outcomes (successful vs. unsuccessful recovery) allow for the reporting of AUC/accuracy, whereas navigation studies use breach rates and grading systems. The reported accuracies are exceedingly high (AUCs ~0.9–0.97), which is atypical for clinical prediction in real-life situations and may indicate that the model is too optimistic due to reliance on a limited number of identical datasets. In such circumstances, conducting multi-center or prospective validation is important. Multiple authors emphasize this necessity: Zheng et al. [68] advocate for additional multicenter testing of their prognostic model, while Li et al. point out the importance of augmenting their dataset to validate V-Net performance [83]. The many study types, ranging from ex vivo feasibility to retrospective clinical cohorts, make direct comparisons more complicated. The examined spine-focused AI studies show encouraging outcomes for implant guiding and predictive analytics. Nonetheless, due to their emerging phase and the utilization of varied methodologies, more testing is needed before applications can be ready for clinical utility.

Moreover, all pertinent studies about AI models and tasks utilized in spine surgery are presented in Table 5.

## 4. Discussion

Artificial intelligence is currently being used at various stages of orthopaedic surgery, encompassing preoperative, intraoperative, and postoperative assessments. In the evaluated studies, the AI appears to be a promising tool for improving both technical and workflow efficiency by analyzing extensive volumes of images, biomechanical data, and clinical information. Nonetheless, the effects of these systems on patients remain uncertain, and an independent standard for validating AI outputs is not consistently available [90]. The principal advantages of AI appear to be in repetitive measurements, image segmentation, landmark identification, implant recognition, and the synthesis of extensive datasets [91]. Nevertheless, the correlation between enhanced technical performance and improved clinical decision-making, patient outcomes, or long-term implant performance is insufficiently established. Table 6 consolidates the prevalent methodological concerns identified in the examined articles, providing a coherent organization of the recurring issues.

The most advanced and evidence-based uses of AI in arthroplasty are currently in preoperative planning and imaging-based evaluation. The use of imaging algorithms can now rapidly produce patient-specific reconstructions and implant models compared to manual or semi-automated methods [40]. This may lead to more uniform templating, reduce interobserver variability, and save the surgeon time in planning. While this could take repetitive tasks off surgeons and make processes more uniform, these systems still require human-generated annotations, curated datasets and expert validation during development [92,93]. However, there is a danger that such AI-generated planning systems will rely too much on purely geometrical or radiographic parameters and not take into account factors such as soft-tissue tension, bone quality, patient-specific biomechanics, surgeon preference, and intra-operative findings.

Overall, AI-driven planning can yield results that are equal to or more accurate than those of experts in implant sizing, alignment measurement, and anatomical reconstruction, and it does so at a significantly faster pace [93]. However, accuracy is not universal; not all studies demonstrate the superiority of AI-guided planning over expert planning or established navigation protocols, and the significance of more precise measurements must be balanced against improved clinical outcomes. The intraoperative application of AI, navigation, or robotics may enhance repeatability and minimize alignment errors; however, thus far, these technologies have not reliably produced clinical advantages in functionality, implant durability, complication rates, or patient satisfaction [94,95]. Future studies must consistently correlate technical endpoints with clinical success measures, including revision rates, dislocation rates, instability, complications, patient-reported outcome measures (PROMs), and long-term implant survival [96].

### 4.1. Integration of AI with Robotic and Navigation-Assisted Orthopaedic Surgery

A notable clinical application of AI is its use in navigation or robotic-assisted orthopaedic surgery. Robotic surgery and computer navigation can augment surgical precision by enhancing the consistency of bone preparation, instrumentation placement, and the execution of surgical procedures [97]. It may enhance these existing platforms through superior preoperative segmentation and planning, prediction of implant dimensions and alignment, identification of anatomical landmarks, and intraoperative plan adjustments based on patient-specific anatomy [97]. In knee or hip arthroplasty, AI assistance in planning may diminish planning duration and enhance consistency, while robotic systems could provide superior reproducibility in execution [94]. In pelvic or spinal surgery, AI-driven image segmentation and trajectory planning may enhance navigation by facilitating the identification of the secure screw corridor [98].

Nevertheless, the advantages provided by AI in conjunction with robotic navigation, as opposed to robotics or navigation independently, remain insufficiently substantiated [99]. Although numerous trials are recording enhancements in technical precision, plan execution speed, or measurement automation, there is scant evidence regarding advancements in patient-centered outcomes, including revision rates, dislocation, instability, complications, functional scores, or long-term implant longevity [97,99]. Consequently, a pragmatic short-term application of AI in orthopaedics would involve decision support for navigation or robotic assistance, rather than fully autonomous surgical systems [100]. Additional research should compare standard surgery, robot-assisted surgery, navigation-assisted surgery, and AI-assisted navigation or robotic surgery, emphasizing clinically significant endpoints with sufficient sample sizes, as well as analyses of cost, workflow, and safety.

It is evident that operational planning for robotic-guided surgery necessitates time for completion and is fundamentally a computational process that can be easily automated. This inefficiency may indicate upcoming developments in software that enable the specification of fundamental alignment principles and limit conditions, subsequently allowing the computer to generate and optimize implant positioning, constrained by anatomical structures, soft tissues, and functional objectives. This would retain the surgeon’s accountability while reducing repetitive technical tasks and the potential for variation.

### 4.2. Limitations of the Evidence Base

Several limitations of the evidence base were identified in the reviewed studies. The majority of included studies were retrospective and derived from single centers, potentially constraining external validity. Numerous studies lacked external validation, indicating that the results of internal validation may exaggerate the system’s actual clinical efficacy [101]. A multitude of technical outcomes were documented favorably in relation to clinical outcomes (e.g., Dice score and accuracy versus revision rates, dislocation rates, complications, functional recovery, or PROMs), with many derived from single-center, internally validated datasets. The studies exhibited variability in dataset reporting; a prevalent issue was insufficient information regarding ethnic diversity, implant type and manufacturer, imaging protocol, imaging equipment manufacturer, class imbalance in images, and discrepancies in ground truth interpretation [102]. The calibration, decision-curve analysis, and failure modes of prediction models were seldom reported [101]. Publication bias exists, favoring positive and technologically sophisticated results in AI research studies [102].

Current AI tools in modern medicine primarily focus on technical accomplishments rather than clinical reasoning, often proving unreliable in atypical scenarios such as misaligned hardware, intricate deformities, revisions, and unconventional implants, where the images significantly deviate from the trained dataset, resulting in lower performance [103]. Consequently, these tools should not be regarded as fully automated solutions, but rather as supportive instruments with potential channels for investigation regarding cost-effectiveness, workflow modifications, training requirements, interpretability, and the medicolegal obligations linked to the utilization of AI tools [100].

Advancements have occurred in postoperative monitoring and risk prediction; however, their translation into clinical practice remains limited [104] and results have occasionally shown adequate discriminatory ability for outcomes such as complications and functional recovery. External validation studies typically reveal inferior consistency of outcomes and present reduced performance at various centers employing different machines, implants, and imaging protocols [105]. The likelihood of a system exhibiting statistical discrimination without clinical relevance is significant for predictive models; for the outcomes to be deemed clinically valuable, they must demonstrate strong performance on calibration curves, evaluated through decision curve analysis.

A significant concern in AI advancements in arthroplasty is that most systems have concentrated on addressing specific technical challenges rather than creating solutions that seamlessly integrate into the patient workflow. New studies frequently emphasize novelty over clinical relevance [106,107]; the majority report enhancements in accuracy, speed, and segmentation precision without addressing bias, unsuccessful studies, or obstacles to implementation and clinical significance of the tool. One should not presume, owing to insufficient external validation and inadequate documentation in studies, that any of these systems have attained genuine clinical readiness.

Consequently, the future of AI systems in surgery is unlikely to be autonomous; rather, they will necessitate effortless integration into clinical and surgical workflows. This will guarantee that AI systems assist the surgeon by managing and organizing information, identifying potential trends, standardizing measurements, and alleviating the cognitive burden associated with each task, while the responsibility and final decision reside with the surgeon. Prioritizing explanation and interpretability is crucial, and a system should ideally indicate when its recommendations may be inapplicable, such as when input images fall outside the training data distribution or when its confidence in the recommendation is below a specified threshold.

Patient-specific digital joint models capable of predicting and simulating surgical outcomes are not prevalent; however, they may significantly contribute to surgical planning, forecasting immediate surgical impacts, and identifying early complications or infections in the future. These systems entail substantial ethical and privacy considerations, necessitate extensive validation testing throughout diverse populations and implant systems, and must consistently predict infrequent yet perilous events. The essential research needed at this juncture is not primarily aimed at validating algorithms but at assessing the beneficial effects of software utilization on patients and outcomes, rather than merely confirming technical viability [108]. Currently, AI in surgery enhances value by minimizing variability and cognitive burden when employed to standardize particular tasks; it cannot supplant the clinician’s expertise and should be regarded as a supportive clinical tool rather than a replacement. Evidence beyond technical validation is now required to confirm improvements in clinical benefit, cost-effectiveness, workflow, and patient-reported outcome measures.

This review has its own limitations as well. It was prepared as a structured narrative review and reported in line with the principles outlined in the Scale for the Assessment of Narrative Review Articles (SANRA) [109]; therefore, no meta-analysis or formal GRADE evaluation of evidence has been conducted. PubMed and Web of Science were utilized to identify pertinent studies, and a PRISMA-style flow diagram was incorporated for transparency; yet other databases such as Embase, Scopus, and CENTRAL were not screened, potentially resulting in the omission of relevant studies. The broad scope across hip, knee, shoulder, and spine surgery limited the ability to perform pooled quantitative comparisons. Nevertheless, the narrative approach allowed comparison of heterogeneous AI applications across anatomical regions, imaging modalities, and clinical tasks.

To strengthen reproducibility and clinical translation, in the realm of orthopaedic AI, future studies should establish AI-specific reporting frameworks: CONSORT-AI for clinical trials [110], SPIRIT-AI for trial protocols [111], and TRIPOD + AI for prediction models [112]. A standard report between all studies, on dataset composition, validation procedures, clinical utility, and performance metrics, will accelerate the safe integration of AI tools into surgical practice.

A further factor limiting clinical translation is the regulatory readiness of AI tools. Although there is an increase in the number of FDA clearances in recent years, the majority of approvals concern radiology and cardiology, with few orthopaedic-specific tools currently accepted for clinical use [113,114].

## 5. Conclusions

The current evidence supports the use of AI as an assistive tool in specific technical procedures within orthopaedic surgery, namely in image segmentation, automated measurements, pre-operative templating, implant identification, component position verification, and select applications for risk prediction. In hip arthroplasty, there was relatively strong evidence to support its use in planning and imaging-based assessment as well as measures for accuracy of measurement, implant sizing, and workflow efficiency. In knee, shoulder, and spinal arthroplasty, applications have been developed although are more heterogeneous in nature and are typically based on retrospective, single-center, or small-cohort studies.

Though the technical performance of AI is often excellent, evidence does not yet support autonomous decision-making in the clinical setting. Many studies reported excellent accuracy values, Dice scores, or angular errors; however, it is less clear whether these improvements translate into clinically significant improvements with regard to revision rates, dislocation, complications, implant survival, functional outcomes, or PROMs. Lack of external validation, inadequate reporting of calibration, data leakage or dataset bias and insufficient failure mode analyses limit its current application in the clinical setting.

Further research should focus on large-scale multi-center external validation studies, pre-operatively splitting patient-level data for training and testing, clearly specifying dataset demographics, providing calibration curves or decision curves with prediction models and failure-mode analysis, and compare results with current robotic or non-AI-based navigation workflows. Clinically meaningful end-points should include operative time, complications, revisions, PROMs, functional improvement, operative cost, and increased burden for learning the systems. As of today, AI should primarily be viewed as a decision support or workflow standardisation tool which complements existing surgical skills.

## Figures and Tables

**Table 1:** Summary of AI-Driven Clinical Applications in Orthopaedic Surgery.

Anatomical Region	Number of Studies	AI Architecture Models	Key Findings	Refs.
Pelvic (Hip Joint & Pelvis)	21	Convolutional Neural Networks (CNNs), U-Net and U-Net variants, Deep Residual Networks (ResNet), Mask R-CNN, Random Forests, Support Vector Machines (SVM), Gradient Boosting, statistical shape modeling integrated with AI	AI was mostly used for preoperative planning, implant positioning, fracture classification, acetabular morphology assessment, and image segmentation. Deep learning models consistently achieved high segmentation accuracy (often >95%) on CT and radiographs. AI improved implant sizing, alignment prediction, and reduction quality, often outperforming manual or conventional planning. Most studies were retrospective and image-based, with limited prospective or outcome-driven validation.	[32,33,34,35,36,37,38,39,40,41,42,43,44,45,46,47,48,49,50,51,52]
Knee	19	CNNs (2D and 3D), ResNet, DenseNet, VGG-based architectures, U-Net variants, ensemble deep learning models, classical ML (SVM, Random Forest)	AI in knee surgery focused on preoperative TKA planning (2D-to-3D reconstruction, component sizing and alignment) and automated postoperative radiographic assessment. Deep learning enabled clinically acceptable 3D reconstructions from standard radiographs and automated templating that can reduce planning time, but accuracy varied and generally remained below expert templating in multicenter settings. AI also supported standardized interpretation of post-TKA radiographs and automated CT-based loosening assessment in specialized protocols. No studies explicitly predicted knee (or patellar) dislocation risk; most evidence was retrospective with limited external validation.	[26,53,54,55,56,57,58,59,60,61,62,63,64,65,66,67,68,69,70]
Shoulder	12	CNNs, U-Net-based segmentation networks, ResNet, DenseNet, transfer learning frameworks, hybrid deep learning–radiomics models	Shoulder AI applications emphasized segmentation (rotator cuff/bone/cartilage on MRI or CT), automated morphometric measurements from radiographs (e.g., CSA/AI), implant identification and postoperative positioning metrics, and outcome prediction. CNN-based segmentation and measurement models achieved near-human accuracy and substantial speed gains, while ensemble networks improved implant classification. Tabular ML models (e.g., XGBoost) showed only moderate individual-level outcome prediction and were mostly internally validated. Overall evidence is heterogeneous and largely retrospective, often based on small cohorts; prospective multicenter validation is needed.	[71,72,73,74,75,76,77,78,79,80,81,82]
Spine	6	3D U-Net and other CNN/U-Net variants (including V-Net), fluoroscopy-based 2D-to-3D reconstruction/navigation models, classical ML classifiers (SVM, logistic regression), neural networks, radiomics-based ML.	Spine AI studies targeted pedicle screw trajectory planning and navigation, vertebral/spinal cord segmentation, and prediction of surgical risks and recovery. Deep learning achieved high segmentation accuracy and near-expert screw trajectory grading in retrospective CT datasets, while fluoroscopy-based AI navigation showed comparable accuracy with potentially reduced radiation in ex vivo workflows. ML/radiomics models reported high AUCs for predicting complications (e.g., vertebral artery injury, adjacent-segment degeneration) and neurological recovery, but were typically trained on small single-center cohorts. Overall, findings are encouraging but heterogeneous, with limited external and prospective validation.	[83,84,85,86,87,88]

**Table 2:** Relevant notes extracted from studies related to AI models designated for the pelvic region.

Anatomical Site	AI Type	Training Dataset	Patient Cohort	Advantages	Limitations	Refs.
Hip joint	AI HIP^®^ software using deep-learning–based segmentation, anatomical recognition, and automatic prosthesis size matching. Neural-network driven 3D reconstruction from CT data.	Not reported	117 consecutive patients (cementless unilateral primary THA). Excluded: severe deformity, prior osteotomy, malunion, Crowe IV DDH, revision surgery.	AI planning is more accurate and stable across all patient subgroups. 2D planning is strongly affected by dysplasia, anteversion, age, and posture errors. AI eliminates issues related to magnification, rotation, and subjective template interpretation. AI planning may enable future integration with PSI, navigation, and robotics.	CT radiation is higher; cost-effectiveness not assessed.	[32]
Hip joint	Artificial intelligence-enabled automated fluoroscopic navigation embedded in the OrthoGrid Systems imaging/navigation platform.	Not reported	420 consecutive primary THAs performed by a single surgeon	AI performs equivalently to trained human technicians. Study highlights importance of fluoroscopic technique and radiographic landmark visibility. AI could reduce staff burden without sacrificing accuracy.	Parallax error likely explains consistent 3° difference between intraoperative and postoperative cup inclination. Single-surgeon, single-implant, single-technique design limits generalizability.	[33]
Pelvis and proximal femur	U-Net architecture with ResNet encoder (ImageNet pretrained)	Imaging from 161 anterior THAs in 146 patients.	Same cohort as above: 146 patients (161 THAs); mixture of pre-op, intra-op, and post-op imaging used to train and test models.	The model performs as well as, or better than, trained human annotators for most clinically relevant landmarks. Model allows real-time intraoperative use (fluoroscopy) and rapid postoperative review. Single-point annotations were chosen to enable fast correction and clinically relevant measurement extraction.	Occasional large outliers exist; surgeon oversight remains important. Inaccurate predictions on structures with poor radiocontrast (ischium, coccyx, SI joints). Current limitations: small dataset, single annotator training, not yet tested live intra-operatively.	[34]
Pelvis and hip joint	Surgeon’s Checklist^®^ AI used within Radlink IAT (image analysis technology)	Not reported	30 patients (86.6% women, median age 58) undergoing primary DAA THA with 3D-IAT. 148 comparison patients (85% women, median age 62) undergoing DAA THA with non-3D-IAT	3D-IAT more accurately predicts functional (standing) component position because it accounts for pelvic tilt change from supine → standing. 3D-IAT performs similarly to robotic-assisted THA in accuracy but with fewer costs, no arrays, and minimal added operative time. Clinical significance may be greatest in patients with abnormal spinopelvic motion.	Non-3D systems fail to correct for pelvic rotation, especially affecting anteversion.	[35]
Hip joint	3D CT-based AI-HIP deep-learning	>2000 CT datasets (various hip diseases)	316 unilateral primary THA patients (April 2019–June 2020) with Tri-Lock femoral stem; mean age 50.7 years; multiple etiologies (majority osteonecrosis)	Acetabular cup complete match: 94.0% vs. 65.2% with manual templating Femoral stem complete match: 87.7% vs. 58.9% with manual templating		[36]
Hip joint	Light Gradient Boosting Machine (LGBM)—main supervised ML model for tabular data Support Vector Machine (SVM)—classification of impingement type Multilayer Perceptron (MLP) + Convolutional Neural Network (CNN)—integrated model combining tabular + imaging data	Primary model: 157 patients Combined image + tabular model: 135 patients	International multicentre prospective cohort 157 adults undergoing primary robotic-arm–assisted THA Mean age ≈ 65 years (range 32–88) Sex: 50.6% female, 49.4% male Impingement found in 64.1% using vROM (bone–bone 52%, implant–bone 41%, implant–implant 7%)	First AI model attempting impingement prediction in THA based on spinopelvic mechanics Spinopelvic parameters (sacral slope, pelvic incidence, cup version, stem version) were key predictors Good ability to detect impingement direction; moderate for presence/type of impingement	Adding imaging did not improve performance due to small dataset and lack of image annotations Requires external validation and larger datasets before clinical adoption	[37]
Pediatric hip joint	3D extension of the SegFormer transformer-based segmentation architecture	98 volumes, from 34 unique pediatric patients	34 newborns (0–6 months) undergoing DDH screening 98 3D ultrasound volumes (4 sweeps per hip)	SegFormer3D outperformed all transformer models and matched or exceeded the best CNN (UNet++) in accuracy. It was significantly more robust to real-world ultrasound variability (blur, scaling, occlusion). Kullback–Leibler divergence (KLD) in the loss function improved global structure consistency, especially for the femoral head.	Mixup augmentation decreased performance; likely due to low SNR of ultrasound and small batch size. Limitations: small dataset, single ultrasound machine + single annotator. Larger multicenter datasets needed.	[38]
Pelvis	Artificial neural network (ANN) using modified 3D SegResNet for CT segmentation Statistical Shape Modeling (SSM) to reconstruct native pelvis Automated ray-casting algorithm for defect quantification	60 CT scans from 115 recruited rTHA candidates	60 patients (mean age 72.1 ± 10 yrs; 28 female, 32 male) Paprosky distribution: 2A (*n* = 2), 2B (*n* = 14), 2C (*n* = 13), 3A (*n* = 14), 3B (*n* = 17) All undergoing revision THA between 2010 and 2023	Pipeline reconstructs 3D acetabular defects from CT in ~15 min Accuracy robust across CT variations and metal artifact reduction May improve surgical planning, implant customization, and rTHA modeling.	Large variability in defect size even within the same Paprosky grade Quantitative metrics (RDV, ADV, DD) do not correlate well with Paprosky classification, signaling limits of radiograph-based grading	[39]
Pelvis	3D neural networks (UNet-based segmentation + point-recognition neural network). Automatic prosthesis matching using a big-data search algorithm + reinforcement learning.	Not reported	53 patients/59 hips undergoing primary cementless THA. Diagnosis: DDH (16), OA (16), ONFH (16), AS (9), RA (2).	AI HIP is highly accurate and dramatically faster than 3D planning. BMI and sex did not influence accuracy. Fully automated planning reduces operator bias and provides reproducible results.	Performance reduced in DDH, especially acetabular cup sizing. AI errors mostly occurred in cases with complex anatomy (ankylosing spondylitis, severe osteophytes, malformed acetabulum, large femoral bowing). AI HIP cannot yet handle certain severe deformities perfectly—needs more training data.	[40]
Posterior pelvic ring	Machine learning + 3D statistical shape modeling (SSM): Regression learners (Gaussian process, SVM, linear regression) predicting principal component (PC) scores for personalized pelvic shape. Classification learners predicting corridor existence.	100 pelvic CTs of uninjured adults 24 anatomical landmarks per pelvis for machine learning predictor features.	20 pelvic CTs from patients with fragility fractures of the sacrum (FFS) (18 women, 2 men; mean age 78.65 ± 8.4 yrs). Used to generate personalized pelvic models (PPMs) and validate implant planning.	First AI-augmented workflow for preoperative planning of transsacral implant placement. AI-assisted planning may significantly improve safety in elderly patients with fragile sacral bone. Personalized models are anatomically accurate when registered to the patient CT. Useful for avoiding malpositioning and identifying safe implant zones.	Corridor variability (size, symmetry, axis direction) is large and requires individualized planning. Workflow took 15–20 min—still time-intensive. Accuracy depends on number and placement of anatomical landmarks.	[41]
Pelvis—superior pubic ramus	Deep learning model: U-Net–based multi-output CNN	103 segmented CTs producing 119 annotated pubic ramus trajectories. 116,400 DRRs generated for training	3 cadaver specimens (from public cadaveric dataset). Used for evaluating sim-to-real corridor and K-wire reconstruction accuracy	First integrated autonomous system for C-arm viewpoint planning + image interpretation + mixed-reality guidance for pelvic fixation. Mixed-reality visualization enhances surgeon awareness. Potential to reduce radiation, OR time, and risk of cortical breach.	Accuracy depends on initial view quality & depth estimation. Narrow bony corridors increase sensitivity to segmentation errors.	[42]
Pelvis	3D UNet deep-learning segmentation Masked multi-step rigid registration (ITK-based) for anatomy repositioning 3D Hough Transform + fast voxel traversal (ray tracing) for screw detection and axis reconstruction	25 preoperative + 27 postoperative CTs (augmented to 520 images)	27 patients (9 male, 18 female), mean age 25 (range 14–33), undergoing PAO between 2018 and 2020	First fully automatic 3D method to quantify all aspects of postoperative PAO outcome: cut placement, fragment repositioning, screw placement. Major improvement over 6-h manual workflows. Most accurate for coarse and fine registration. Screw quantification robust even without isolated screw masks.	Training requires extensive manual labelling; generalisation to other surgeries is future work. Osteotomy plane prediction is the most challenging due to callus formation and irregular fracture-like cuts. CT-based approach implies radiation but compatible with future low-dose imaging.	[43]
Hip joint	AI-HIP Version 1.0 software (Beijing Changmugu Medical Technology): Deep learning–based G-NET neural network	Not reported	109 patients undergoing primary THA for unilateral ischemic necrosis (55 AI group, 54 2D group). Baseline characteristics (age, BMI, Ficat stage, pre-op LLD, eccentricity, VAS, Harris) were statistically comparable	AI-HIP improves: Implant size prediction Surgical efficiency (shorter time, fewer trial components) Postoperative LLD Bleeding and hospitalization time Early functional recovery (Harris score).	Limitations: limited prosthesis library (DePuy only), single disease (ischemic necrosis), limited imaging parameters, no external validation.	[44]
Right hip joint	Not reported	Not reported	Single patient, 66-year-old female, multiple prior THA surgeries, large acetabular and proximal femur bone defects (Paprosky-type features described).	AI allowed accurate planning of cup size, position, and augment design. 3D-printed augments ensured precise fit to bone defects, improving early stability. AI-generated test models enabled rehearsal before surgery. Case resulted in excellent short-term clinical improvement and stable early fixation.		[45]
Hip joint	ChangmuGu 3D system: deep convolutional neural networks model	Not reported—system is a previously trained and validated machine-learning model	92 patients (49 AI-3D, 43 2D X-ray). All Crowe type II–IV. Follow-up: 24 months. Baseline variables all statistically equivalent.	AI-assisted planning improves prosthesis sizing, component positioning, LLD correction, operative time, and blood loss	AI implementation does not change 24-month functional scores or implant survival. CT radiation dose, cost, and workflow complexity remain barriers to broad adoption.	[46]
Hip joint	Artificial Neural Network (ANN) Shallow feed-forward network 2 hidden layers, 10 total nodes Hyperbolic tangent activation (hidden layers), linear output	17 healthy subjects 170 ANN training iterations	Same as training cohort (healthy population only). No clinical patients included.	ANN predicted hip frontal moments best (R^2^ > 0.90). Sagittal and frontal angles were moderately accurate;	Sagittal moments more difficult. Peaks consistently underestimated vs. gold-standard. Variability across subjects—adduction angle hardest to generalize. Gold-standard OpenSim moments likely inflated due to center of pressure (CoP) modeling error.	[47]
Femoral head	3D Dynamic U-Net segmentation model (primary model)	63 hips from 56 ONFH patients (JIC stage 1–2)	Same 63 hips from 56 patients (20 men, 36 women; mean age 45 years; range 14–75). All pre-collapse ONFH	3D model significantly outperforms 2D model for complex necrotic shapes. Increasing training data beyond ~30 cases did not meaningfully improve accuracy unless outlier cases included. Excellent interobserver agreement (surgeons r = 0.99; AI vs. surgeon B DC = 0.84). Useful for volumetric Steinberg classification and automated collapse-risk evaluation.	Error cases involved large lesions extending into the neck. No external validation; relies on a single surgeon’s definition of necrotic	[48]
Femur	3D deep learning–based segmentation using nnU-Net Statistical Shape Model (SSM) GT-ICNC and piriformis-ICNC reference axes	70 independent CT scans of bilateral femurs	63 participants, 126 femurs 35 men, 28 women Age: 52.0 ± 14.7 (range 20–75) Excluded prior trauma/surgery Final dataset used for comparing automated vs. manual FNA methods.	Automated method avoids human landmark variability. Two automated axis definitions (GT-ICNC, piriformis-ICNC) yield identical results (ICC = 1). Strong consistency with Murphy & Reikeras manual techniques. Lee method systematically underestimates FNA → explains differences. Automated 3D approach more robust than 2D CT slice–based manual methods.	Limitations: Not tested in severe deformity, pediatric cases, or extreme pathologies; SSM trained on population-specific anatomy; generalizability requires further testing.	[49]
Pelvis	Two-stage, multi-task deep learning framework: Stage 1: Dual-task shared-encoder network for global bone segmentation + landmark detection. Stage 2: Edge-enhanced multi-task network for refined acetabular segmentation + edge detection.	81 CT scans total: 31 diseased hips (ONFH, OA, DDH, femoral neck fracture, bone tumor). 50 healthy CT scans from the COLONOG database. Threefold cross-validation used.	31 patients, age 33–87 (mean 62), 16 males/15 females. Disease distribution shown in Table 1 of article (e.g., OA = 14, DDH = 7, ONFH = 3, FNF = 6, BT = 1).	Handles difficult diseased cases (joint space narrowing, weak bone boundaries, deformity). Multi-task learning allows segmentation and landmark tasks to reinforce each other. Edge-enhancement in Stage 2 dramatically improves acetabulum boundary precision. Computational efficiency: ~10.9 s for full workflow (half the time of U-Net). Supports accurate acetabular cup design and THA planning; addresses limitations of single-task models.		[50]
Spinopelvic	Back Propagation Neural Network (BPNN)	Training set = 80% of 145 volunteers (approx. 116)	145 healthy adults (51 M/94 F), age 19–29	BPNN outperformed multilinear regression, elastic net, and SVR; strong correlations were identified between standing and sitting spinopelvic parameters.	Limited by young healthy cohort and manual measurements	[51]
Pelvis and proximal femur	Transformer-based surgical phase recognition (SPR) model with: U-Net encoder–decoder for spatial annotations (segmentation + landmarks) Transformer sequence model for temporal reasoning Multi-task learning for: corridor, activity, view, and frame classification	Simulation-based dataset using 337 annotated CT scans.	Cadaver study: 1 lower torso specimen with 5 screw insertions. Total images: 257 real intraoperative X-ray images. Labels assigned based on surgeon narration	First framework for SPR using X-ray images. Sim-to-real transfer is operational. Additional supervision (corridor/landmark/tool segmentation) improves representation learning.	A gap remains between synthetic and real images. Model struggles with visually similar views (e.g., obturator vs. teardrop). Clinical variability (surgeon preference, C-arm path) introduces ambiguity	[52]

**Table 3:** Relevant notes extracted from studies related to AI models designated for the Knee Joint.

Anatomical Site	AI Type	Training Dataset	Patient Cohort	Advantages	Limitations	Refs.
Lower limb	Multi-network deep-learning pipeline (leg separation CNN + landmark CNN + 2D → 3D U-Net reconstruction) integrated with genetic algorithm automated HTO planning	175 CT patients (segmented tibia + hip/knee/ankle centers). DRRs generated and augmented to 525 EOS-like pairs	52 real HTO patients used to evaluate feasibility of reconstructed models for automated planning	Handles superimposed legs via dedicated separation network (improves Dice). Clinically acceptable 3D reconstruction accuracy and mechanical axis alignment. Enables fully automated HTO planning with small correction-angle and axis-position errors.	Tibial slope accuracy insufficient due to incomplete tibial plateau visualization on radiographs. Needs training on real EOS + domain adaptation to reduce synthetic-to-real gap.	[53]
Distal femur	Automated landmark identification via: (1) Neural Network (NN), (2) Statistical Shape Model (SSM), (3) Geometric approach (GA)	101 patients/202 distal femurs (80% train, 20% test on non-osteophyte femurs); osteophyte femurs used for robustness testing; 2 raters for ground truth	Same as training cohort: 101 Japanese THA patients (202 femora total)	NN and SSM achieve accuracy comparable to manual landmarking (low deviation) with high success rates. Robustness generally preserved even with osteophytes. Suitable for high-throughput research and preop workflows.	GA is less accurate and fails more often in osteophytic/deformed femora. NN requires CT and sizable training data; SSM requires segmented meshes. Single-ethnicity dataset, limited deformity spectrum, only two-rater ground truth.	[54]
Knee joint	Suite of 12 CNN algorithms for radiograph QA, landmark/angle regression, and interface anomaly detection (commercial: Bianka/Deemea)	39,751 radiographs (22,759 patients): large multi-task annotation sets; 60/20/20 split	60 radiographs evaluated; senior surgeons labeled with/without AI assistance	High QA accuracy (95–99.9%). Angle prediction error ~1.75°, comparable to senior surgeons. Anomaly detection strong (AUC ~0.94). Improves surgeon accuracy (+5%), sensitivity (+12%), and agreement (kappa ↑ intra +0.17, inter +0.10).	Single-center/limited heterogeneity; needs multicenter validation and broader imaging variation.	[55]
Knee joint	Deep-learning 2D-to-3D reconstruction (RSIP XPlan.ai™—RSIP Vision, Jerusalem, Israel) using neural networks + statistical modeling + 3D calibration	>1000 pathological knee samples (training)	18 TKA patients (real clinical anatomies)	Sub-millimetric global and local accuracy (RMSE ~0.9 mm surfaces; ~0.5 mm landmarks). Cut-plane contours accurate (<1 mm RMSE). Angular deviations close to human baseline variability → potential CT alternative for robotic/PSI workflows.	Requires standardized AP/lateral radiographs + calibration jig. Small clinical cohort (*n* = 18); generalizability uncertain.	[56]
Knee joint	Multi-task deep learning (segmentation + keypoints + line detection) with GradNorm balancing; intra-op guidance with real-time adjustment	Pre-op dataset: 38 radiographs with segmentation masks, keypoints, line annotations (MPFL/ACL/PCL tasks)	Intra-op test: 15 trauma cases; 3 ACL cases unusable due to segmentation failure	Multi-task learning improves accuracy vs. single-task; tasks reinforce each other. High pre-op precision (drill point < 2.9 mm; k-wire angle < 0.75°). MPFL performance meets clinical requirements; supports intra-op adjustment and registration.	Intra-op performance degrades with metal overlap/depth ambiguity; tibial ACL points hardest. Segmentation failures occurred (ACL cases). Limited scaling/translation of px-to-mm for some tasks (PCL incomplete).	[57]
Knee joint	AI-KNEE 3D preoperative planning (proprietary G-NET deep learning; commercial pretrained)	Not reported	60 KOA primary TKA patients (30 AI vs. 30 2D), same team + same implant manufacturer	Higher prosthesis size matching (femur 90% vs. 66.7%; tibia 86.7% vs. 60%). Fewer alignment outliers (VCA, HKA). Better early functional outcomes (WOMAC, AKS at 3–12 months).	Single-center, small sample, short/mid-term follow-up. Implant library limited (single manufacturer).	[26]
Patellofemoral joint	Two-stage deep learning regression: ResNet50 aligner + seven ResNet50 patch models; SimCLR/RadImageNet pretrained	483 patients; 14,652 annotated axial CT images (healthy + OA/arthroplasty cohort)	Same combined cohort; train/val/test 329/59/95 patients	Large CT-trained model for patellofemoral anatomy. High landmark precision (patch models > 93% within 0.40 cm). Strong agreement for multiple PF measurements; robust across healthy and OA knees. Enables large-scale anatomy studies and planning/implant design.	Axial CT only; assumes full anatomy present in slices. Dataset imbalance (OA/KA > healthy).	[58]
Tibia	nnU-Net segmentation (2D & 3D), final: Cortex 3D nnU-Net for implant/bone segmentation enabling loosening metrics	Segmentation training: 25 valgus-loaded CT scans (20 cadaver + 5 patient) with manual labels	Cadaver: 20 CT pairs; Patient: 77 CT pairs (asymptomatic/symptomatic/loose); Reproducibility: 10 unloaded CT scans	DL segmentation replaces semi-automatic workflow without loss of diagnostic separation. Excellent agreement with manual (ICC ~0.99 cadaver; strong inter-operator ICC 0.92–0.99 in patients). 3D model outperforms 2D; cortex mask optimizes downstream registration.	Sometimes underestimates absolute displacement values. Loose vs. fixed not separable in patient cohort (same as manual). Full-bone masks caused registration failures → segmentation choice impacts pipeline.	[59]
Femur	AI JOINT™ preoperative planning (deep-learning segmentation + landmark recognition + DL + RL prosthesis matching) used for ligament-safe osteotomy simulation	Not reported (pretrained commercial system)	Single healthy volunteer (25 yrs); deformity simulation set	Demonstrates AI-guided visualization of ligament proximity and osteotomy safety. Shows small controlled alignment adjustments (±3°) + residual HKA can avoid collateral ligament injury in most deformities. Highlights deformity location effect (distal deformities highest risk).	Not an ML validation study: no typical accuracy metrics, no clinical patient cohort. Findings based on simulated deformities and one volunteer → limited clinical generalizability.	[60]
Knee joint	ML regression for operative time prediction (Linear/RF/CatBoost; CatBoost best) using demographics ± CT 3D data	1061 robotic-assisted TKAs (2016–2019), two surgeons/two centers; CV + test split	Same 1061 retrospective cases	Predicts OR time well; CT morphology improves performance (more predictions within 5–15 min; higher R^2^). Identifies key predictors (surgeon ID, weight, osteophyte volumes). Useful for OR scheduling and reducing delays/cancellations.	Only two surgeons; external validation lacking. Limited variable set (e.g., bone quality not included).	[61]
Knee joint	Multi-step DL templating: CNN landmark detection + Swin Transformer segmentation + HRNet landmark model	13,281 knee radiographs for training; 2302 val/test; dedicated segmentation/landmark subsets	81 TKA surgeries (72 patients) for clinical evaluation	Fast (≈49 s) and reasonably accurate within ±1 size (~89% femur/tibia). Implant position error ~3–4 mm; alignment error < 2° (except femoral sagittal). Not affected by age/sex/BMI; under-sizing strategy may reduce overhang risk.	Manual templating still better for ±1 size in this cohort. Segmentation struggles in severe deformity. Single-ethnicity, single-implant model; external validation lacking.	[62]
Knee joint	LSTM (RNN) injury detection using engineered biomechanical features from broadcast video; compared to FCNN	210 video clips (129 athletes), ~32 k frames; imbalanced classes	Professional athletes across 11 sports (67% male)	First in-game ACL injury detection from broadcast footage; 3D pose from single view feasible. Good discrimination (ROC AUC ~0.88) and interpretable biomechanical signals. Human reviewers improved with AI-derived pose/signals.	Class imbalance and many exclusions due to visibility. Manual supervision needed for tracking. Reduced performance for female athletes.	[63]
ACL	Transfer-learning DCNN (Inception-v3 pretrained on ImageNet) for ACL tear classification on MRI	MRNet dataset; 1370 MRI knee images (70% train/val)	30% MRNet test set (411 images)	High reported classification metrics (accuracy/precision/recall/specificity ~95%+). Transfer learning effective with limited dataset; preprocessing improves consistency.	Single dataset evaluation; generalizability across institutions/scanners not proven. Task is classification only (no localization/tear grading).	[64]
Hip–knee–ankle pathway (HKAA)	Three-stage pipeline: VGG16 + XGBoost slice selection → 2D TransUNet segmentation → OpenCV measurement extraction (27 metrics)	Not specified	1352 pre-TKA CT patients (large non-industry dataset)	Large-scale CT-based anatomic phenotyping; high segmentation performance (Dice > 0.94, IoU > 0.95). Automates extraction of 27 metrics; identifies substantial “anatomic outlier” subgroup (~31%). Highlights morphology variability not captured by radiographs → supports personalized planning.	Training dataset details not stated. Thresholding/outlier definition may be methodology-dependent; needs external validation and clinical outcome linkage.	[65]
Knee joint	Unsupervised ML gait phenotyping (PCA + MDS + hierarchical clustering)	Gait waveform dataset (134 pre-TKA; 105 with 1-yr follow-up)	Severe knee OA (mostly KL 3–4); able to walk without aids; data collected 2003–2016	Identifies 4 clinically meaningful phenotypes; sex strongly influences cluster membership. Links phenotype to postoperative biomechanical improvement (low-functioning clusters improve most). Potential tool for expectation-setting and personalization.	Moderate clustering strength (silhouette ~0.37). Retrospective/older acquisition era; generalizability to modern cohorts uncertain.	[66]
Knee joint	CNN templating using ResNet-101 classification (implant size prediction) on AP + lateral radiographs	714 patients (2010–2014), 1412 radiographs augmented; 80% train	20% test split	High sizing performance (micro F1 high; ±1 size near-perfect). Works using radiographs only (no scaling/demographics). Lateral views performed best.	Single-surgeon/single-ethnicity dataset; generalizability uncertain. Pure classification (no 3D planning/pose).	[67]
Knee joint	DL (LSTM) + ML ensemble predicting knee kinematics/forces; training data from musculoskeletal multibody model based on one patient	Simulated training generated from one subject’s experimental motion data	Not specified	DL most accurate for most kinematics and all contact forces; excellent correlations. Potential to define safety calibration zones for TKA planning/robotic guidance.	Single-patient simulation limits validity and generalizability; requires multi-subject experimental datasets.	[68]
Lower limb	ML/DL comparison for gait-based classification (logistic/LASSO, XGBoost, InceptionTime, FCN, transfer learning + augmentation)	Dataset 1: GaitRec (*n* = 2295). Dataset 2: PFPS (*n* = 31). Nested CV + subject-level CV	Not specified	Shows XGBoost competitive on moderate/large datasets; DL can match when data sufficient. Demonstrates augmentation helps large datasets; TL from image models beneficial.	Small datasets cause unstable DL performance; augmentation may harm small sets. External validation and standardization across gait labs remain challenges.	[69]
Femoral intercondylar notch	3D CNN segmentation (best: SegResNet) + Statistical Shape Modeling (PCA) for notch morphology on MRI	109 MRIs collected; 100 ACL-injured included; DL set augmented to 276 volumes (75% train, 20% validation, 5% test)	100 ACL-injured patients (31F/69M; mean age ~31)	Good 3D segmentation (DSC ~0.88) and rapid volume computation (seconds vs. minutes). SSM quantifies key variability (size/shape/height PCs) and demonstrates sex differences in notch volume. Clinical relevance for tunnel placement, graft sizing, notchplasty planning, impingement risk.	Only ACL-injured cohort (no healthy controls for normative modeling). Single-center timeframe; needs external validation across scanners/protocols.	[70]

**Table 4:** Relevant notes extracted from studies related to AI models designated for the shoulder area.

Anatomical Site	AI Type	Training Dataset	Patient Cohort	Advantages	Limitations	Refs.
Proximal humerus	Deep learning semantic segmentation (DeepLab v3+ + Inception-ResNet-v2) for fracture fragments + Monte Carlo simulation + decision tree for automatic virtual reduction	5,619,032 CT images (60/20/20 split) with 5-fold cross-validation	20 Neer 3–4 part PHF patients with anatomic post-op reduction validated on post-op 3D CT	Automated reduction outperformed manual reduction in shape similarity (higher DSC/IoU) and surgeon-rated quality (RQS) while reducing planning time massively (~50 s vs. ~58 min). Removes need for time-intensive manual segmentation and reduction. Monte Carlo + decision tree may avoid ICP misalignment issues in complex fractures.	Small clinical cohort (*n* = 20). Reference used post-op CT rather than mirrored contralateral anatomy. No assessment of downstream clinical outcomes (only planning quality). Potential concern: reduced hands-on learning for trainees.	[71]
Shoulder joint	nnU-Net (2D & 3D U-Net) MRI segmentation with secondary labeling to reduce false positives	34 MRIs train (60%), 11 tune (20%), 11 internal test (20%); +10 external MRIs multi-institution (Philips/Siemens; 1.5T/3T)	Internal test: 11 MRIs; external DSC eval: 10 MRIs from multiple institutions	Secondary labeling reduces false positives (esp. LHB tendon), improving tendon DSC. 3D U-Net improves anatomical continuity vs. 2D; fast inference (10–30 s) vs. manual (~40 min). Accuracy comparable to intraobserver variability and better than interobserver variability. Tear size did not significantly affect segmentation performance.	Very small training/validation sets. Limited external test size (*n* = 10). Generalization beyond included scanners/protocols still uncertain.	[72]
Shoulder joint	Supervised ML outcome prediction: XGBoost regression + classification for PROMs and MCID/SCB	66.7% of 5774 shoulder arthroplasty cases (2153 aTSA; 3621 rTSA)	Remaining 33.3% of same dataset (broad diagnoses; aTSA mean ~66 yrs; rTSA ~72 yrs)	Minimal 19-feature model performs nearly the same as 291-feature model (efficient for deployment). Good performance for PROM and ROM prediction (similar MAE between minimal vs. full). Strong ability to classify MCID achievement at 2–3 years (high accuracy/AUROC range reported). Practical for clinical decision support and risk stratification.	Registry-style limitations: model performance depends on data quality and follow-up completeness. External validation outside the source dataset not described here. Adds implant/glenoid anatomy only marginally—may limit perceived benefit of CT-heavy workflows.	[73]
Shoulder joint	3D CNN encoder–decoder segmentation (CEL-UNet) + 3D CNN multi-task classifier (Arthro-Net) on CT	571 CT scans (after excluding 36 with metalwork): 410 train, 71 val; 90 test	90 CT scans test set covering wide GH OA severity spectrum	Very high segmentation + reconstruction accuracy (Dice ~0.98–0.99; low RMSE; robust even in severe OA). Maintains native CT resolution (reduces interpolation artifacts). Fast end-to-end runtime (<15 s) and captures osteophytes/narrow joint spaces well. Oriented toward PSI-based planning automation.	Excluded metalwork scans → may limit applicability in post-op/revision scenarios. Needs external multicenter testing for true generalizability.	[74]
Shoulder joint	Proprietary ML classification models (OBERD–Universal Research Solutions) predicting ASES improvement classes; compared models with/without CT morphology and latent ASES variables	Closed dataset: all 472 shoulders used for training (no external validation)	472 primary GH OA patients (431 TSA, 41 RSA), mean age 68, 56% male	Best performance when combining latent ASES variables + CT morphology + demographics (morphology and PROM “latent” features are complementary). Identifies influential predictors (ASES items, Walch type, pain, cuff fatty infiltration). Suggests longer follow-up windows may improve prediction stability.	No external testing; risk of overfitting (closed dataset). Retrospective, single-surgeon design limits generalizability. Reliant on preop ASES latent variables, which may reduce automation if PROM capture is incomplete.	[75]
Deltoid muscle	SwinUNETR CT segmentation of deltoid + XGBoost outcome prediction using deltoid morphology (radiomics)	Segmentation: 78 labeled CTs train + 20 test. Prediction: 1057 arthroplasty patients’ preop CT + outcomes	1057 shoulder arthroplasty patients (799 rTSA, 258 aTSA) with preop CT + ≥2-year outcomes	Large cohort linking deltoid morphology to outcomes; image-based models reduce prediction error for ROM vs. non-image models. Identifies high-value deltoid features (shape/volume descriptors). Enables more automated decision support (potentially reducing reliance on manual PROM inputs). Strong deltoid segmentation performance reported.	Radiomic features sensitive to CT reconstruction kernel (affects generalizability). Needs external validation and harmonization across scanners/protocols. Adds complexity (segmentation + radiomics) compared with tabular-only models.	[76]
Shoulder joint	U-Net–like CNN with EfficientNet-B3 encoder + view classifier (ResNet-18) for automated CSA and AI measurement on AP radiographs	MURA v1.1: 1004 train + 174 val AP radiographs; single-expert landmark annotations; separate view classifier	93 independent test radiographs	Automates CSA and AI measurements with errors within/at human interobserver variability ranges. Heatmap regression improves robustness vs. direct coordinate prediction. Potential for PACS integration and large-scale research measurement extraction.	Single annotator; no demographic stratification available. Some radiographs not true AP (measurement sensitivity). External validation across institutions not shown.	[77]
Proximal humerus	EfficientNet-Lite0–based model + CRF-RNN post-processing; Hausdorff-distance loss for boundary-sensitive anatomic neck detection	62 humeri (37 healthy, 25 arthritic): 80% train; ground truth from surgeon points; 3D models segmented using separate in-house CNN trained on 180 humeri	Same 62 CT-derived humeri; test set 14 (8 arthritic, 6 healthy)	Directly detects the anatomic neck (not just resection plane); maintains performance despite osteophytes. Low centroid and angular errors; useful for implant positioning metrics (retroversion, neck–shaft, resection height). Uses 3D models (potentially more robust across CT scanners). Included in an open-source shoulder Python package.	Small dataset (*n* = 62) and no external validation. Diverse anatomies remain challenging; arthritic cases show higher error. HD still sizable; downstream surgical impact not validated clinically.	[78]
Shoulder joint	Ensemble DL implant classification (IMFC-Net): modified Inception-V3 + modified MobileNet-V2 + MLP; Convolutional Pooling + Rotational Invariant Augmentation	597 post-op shoulder radiographs across 4 manufacturers; 10-fold CV with RIA augmentation	597 patients (one post-op shoulder X-ray each)	Strong implant classification with RIA improving robustness to orientation variability. CP blocks and sequential training improve feature extraction and avoid “model dominance”. Outperforms several baseline CNN families; Grad-CAM suggests discriminative implant feature learning.	Class imbalance and low inter-class variability make classification difficult. Closed-world setting; generalization to unseen implant models and non-AP views remains a challenge.	[79]
Shoulder joint	Dense Residual Ensemble Network (DRE-Net): modified ResNet-50 + modified DenseNet-201 + shallow concatenation; includes RIA	597 implant radiographs (same 16 models, 4 manufacturers); 10-fold CV; heavy augmentation (~36×)	597 patients (one post-op X-ray)	Ensemble learning improves classification over single backbones; RIA crucial for orientation variance. Addresses closed-world and discusses open-world implant identification scenarios. Demonstrates value of deep features vs. classical PCA + KNN.	Class imbalance, heterogeneous sources, reliance on AP views. Closed-world training limits real-world performance estimates for unseen implants.	[80]
Shoulder joint	U-Net segmentation + automated geometric measurement (line-fitting/annotation) for post-rTSA radiographic metrics; GUI-integrated	417 post-op rTSA radiographs (4 manufacturers), split by patient and implant type; test set 85	17 primary rTSA patients	High agreement with humans for 5 measurements (ICCs ~0.90–0.96) with small MAE; very fast runtime (~2 s vs. >10 min). Segmentation quality comparable to observer–observer agreement. Clinical usability enhanced via GUI integration.	Some implant-specific visibility issues (e.g., DePuy Delta Xtend baseplate affects certain measures). Glenoid neck segmentation suboptimal (though measurement robustness maintained). Small clinical patient cohort (*n* = 17) despite larger image dataset.	[81]
Rotator cuff muscles	DeepLabV3+ (ResNet50) slice-wise CT segmentation for rotator cuff muscles with longitudinal assessment	Training segmentation set: 53 patients (32 train/11 val/10 test) with slice augmentation	172 TSA patients with longitudinal CT: pre-op (162), 2-year (152), 5-year (121) usable scans	High segmentation accuracy across timepoints; enables efficient longitudinal muscle health monitoring. Handles beam-hardening/metal artifacts reasonably; outperforms 2D U-Net significantly. Performance close to intra-reader variability → supports reliable volumetrics at scale.	Single annotator ground truth; modest segmentation training cohort; no external validation. Poor-quality scans excluded; manual landmark selection still needed.	[82]

**Table 5:** Relevant notes extracted from studies related to AI models designated for the spine.

Anatomical Site	AI Type	Training Dataset	Patient Cohort	Advantages	Limitations	Refs.
Thoracolumbar spine (T8–T12 and L1–L5)	Improved V-Net deep learning CT segmentation for vertebral 3D reconstruction (compared vs. U-Net, V-Net, CNN)	Not reported	106 patients (128 vertebrae) with osteoporotic thoracolumbar compression fractures: 53 PKP vs. 53 PVP; 63M/43F	Higher DSC and lower Hausdorff distance than comparator networks → better 3D reconstruction continuity/detail. Enables improved evaluation of vertebral injury and post-treatment morphology. Clinical comparison suggests PKP better than PVP for height restoration/kyphosis correction/pain relief/early recovery (within this cohort).	Training dataset not described. Small cohort and limited to thoracolumbar osteoporotic fractures. Network performance analysis reported as limited/insufficient.	[83]
Lumbar spine (L1–L5)	X23D AI-based fluoroscopy 3D reconstruction for navigation (no intraop CT/registration)	Not reported	6 cadaveric torsos; 5 spine surgeons placed 10 screws each (5 X23D, 5 control)	Supports navigation without intraoperative CT or registration → simpler workflow. Pedicle screw breach rates comparable to control; reconstruction extremely fast (~80 ms/vertebra). Radiation exposure comparable or lower than standard fluoroscopy workflow (in reported analysis). Better surgeon-rated workload/usability than 2D fluoro and existing navigation (NASA-TLX).	Very small cadaver-only study; not clinical patients. Occasional reconstruction failure (1/30 vertebrae). Prototype lacked a dedicated spine-mounted tracker; generalizability uncertain.	[84]
Deep cervical paraspinal muscles (multifidus, semispinalis cervicis)	SVM predictive model for early adjacent segment disease (ASD) after ACDF using muscle morphometrics	62 patients total used for model building (32 early-onset ASD; 30 matched controls)	Same 62 adults (mean age 52.4 ± 10.9) undergoing two-level ACDF (C3–C5/C4–C6/C5–C7); ASD assessed ≤6 months	High reported predictive performance (accuracy 96.7%, AUC 0.97). Identifies muscle asymmetry and lean CSA features as key predictors, outperforming demographics/radiographs. Enables preop risk stratification and supports rationale for targeted prehab/rehab focused on muscle health.	Small single-cohort dataset; no external validation. Short follow-up window (early ASD only) and possible measurement variability in muscle features.	[85]
T12–S1 (lower thoracic, lumbar, sacral spine)	3D U-Net CT segmentation + morphological algorithm for automated pedicle screw planning	160 clinical cases	70 clinical patients	High segmentation Dice (~0.95 across T12–S1). Very high planned screw “accuracy” by Gertzbein–Robbins (98.8% Grade A; no Grade C–E) with low facet violation rates; good observer agreement. Fast runtime (≈26 s segmentation; ≈2 s per screw plan). Uses preop CT (avoids CBCT artifacts/time burden).	Excludes severe deformities and major degenerative changes (limits real-world coverage). Needs multicenter external validation; real intraoperative execution accuracy not established from planning alone.	[86]
C2 (Axis) vertebra	ML risk prediction for C2 pedicle injury (tested LR, SVM, GBM, NNet, XGBoost, KNN, AdaBoost, CatBoost; best = Neural Network)	280 CTA scans (train 197; validation 83)	280 patients total: 98 injury vs. 182 non-injury	Best NNet shows strong discrimination and calibration (AUC ~0.93 train/validation) with favorable decision-curve net benefit. Identifies key anatomic risk factors (e.g., pedicle diameter, HRVA/IAVA, VAD, etc.) with SHAP interpretability → useful for preop planning and risk counseling.	Single-center, modest sample size; no external validation. Dependent on measurement quality/subjectivity of CTA-derived variables; potential overfitting in some alternative models noted.	[87]
Cervical spinal cord	ML radiomics + clinical prediction (SVM/RF/Extra Trees etc.; best radiomics SVM; best combined radiomics + clinical)	101 patients	25 test patients	Combined radiomics + clinical predictors substantially improves performance (accuracy 0.895, AUC 0.967 vs. radiomics-only). Narrowest-segment feature extraction is optimal.	Small independent test cohort (*n* = 25). Generalizability depends on consistent segmentation and imaging protocols;	[88]

**Table 6:** Methodological limitations identified across the included AI studies.

Domain	Main Issue Identified	Relevance
Study design	Most studies were retrospective	Limits causal and clinical interpretation
Dataset origin	Many studies used single-center datasets	Reduces generalizability
Validation	External validation was uncommon	Increases the risk of overestimated performance
Data splitting	Patient-level data splitting was inconsistently reported	Increases the risk of data leakage
Dataset bias	Limited reporting of ethnicity, implant type, scanner type, and imaging protocol	May contribute to algorithmic bias
Prediction models	Calibration and decision-curve analysis were rarely reported	Limits interpretation of clinical risk and utility
Clinical endpoints	Few studies linked AI outputs to revision, complications, PROMs, or implant survival	Limits assessment of clinical relevance
Workflow	Time, cost, training burden, and implementation data were inconsistently reported	Limits assessment of real-world adoption

## Data Availability

No new data were created or analyzed in this study. Data sharing is not applicable to this article.
